# Migraine and Mood in Children

**DOI:** 10.3390/bs11040052

**Published:** 2021-04-14

**Authors:** Parisa Gazerani

**Affiliations:** 1 Department of Life Sciences and Health, Faculty of Health Sciences, Oslo Metropolitan University, 0130 Oslo, Norway; parisaga@oslomet.no; 2 Department of Health Science and Technology, Faculty of Medicine, Aalborg University, 9229 Aalborg E, Denmark; gazerani@hst.aau.dk

**Keywords:** migraine, mood, headaches, pediatrics, children, multidisciplinary, biofeedback, relaxation, cognitive-behavioral therapy, lifestyle

## Abstract

Migraine is one of the most prevalent headache disorders in children and negatively influences their quality of life. Physical, social, and school functioning are negatively affected. Mood changes are common in migraineurs and may happen before, during, or after a migraine headache. Children with migraine are not exempt from mood swings. The majority of mood changes occur during the prodromal phase, manifesting as a psychobiological response, e.g., difficulty thinking, trouble concentrating, irritability, higher or lower energy, confusion, and depression. A bi-directional relationship between migraine and mood has been proposed, but mechanisms are not clear. Collectively, a maladaptive stress response has been suggested to explain the inability to balance homoeostatic changes when facing various stressors. Recognizing mood changes and monitoring mood patterns in children with migraine, for example, by various apps and so-called mood monitors, is valuable for better management. A multidisciplinary intervention has been recommended to reduce migraine disability, improve coping strategies, and reduce chronification risks in children with migraine. Pharmacological and non-pharmacological strategies are both available and effective. Biofeedback, relaxation, and cognitive-behavioral therapy yield positive outcomes in pediatric migraine. Developing healthy lifestyle habits (diet, exercise, sleep) also seems to improve migraine in this population.

## 1. Introduction

Children with migraine present mood swings that may happen before, during, or after a migraine headache. The majority of mood changes occur during the prodromal phase, manifesting as a psychobiological response, e.g., difficulty thinking, trouble concentrating, irritability, higher or lower energy, confusion, and depression. A bi-directional relationship between migraine and mood has been proposed, but mechanisms are not clear. The goal of this review is to examine the existing literature on migraine and mood in children and adolescents, and to provide an up-to-date picture of what is known and what is lacking. Identifying research gaps could serve as a starting point for future clinical implications and optimal guidelines. The following search terms were used: “headache” OR “migraine” and related terms, AND “children”, “childhood”, “child”, “adolescents”, “adolescence”, “young”, “mood”, “mood swings”, “psychiatric comorbidities”, “behavioral”, “behavioral treatment”, “behavioral therapy”, “pharmacological treatments”, “non-pharmacological treatments”, “cognitive-behavioral therapy”, and “psychological intervention”. MEDLINE, Web of Science, and Google Scholar databases were searched to gain adequate and efficient coverage. In addition, reference lists of identified papers were judged and used wherever relevant. Additional peer-reviewed literature identified through citations of the key articles was also included. The search was unlimited in start time, but limited to the English language.

### 1.1. Migraine 

Migraines have been generally avoided by the academic medical community. It was thought that migraine did not occur in children until after puberty. In the early 1950s, the first attempt was made to define migraine in children [1]. Afterward, several proposals were introduced to the field.

The current classification of migraine is presented by the International Headache Society [2]. Migraine diagnostic criteria look similar to those defined for adult migraine [2]; however, in pediatric migraine, some unique features are presented, mainly, a shorter duration of headaches (can be as short as 2 h) and bilateral localization. A short duration of 1–2 h is not uncommon in prepubertal children with episodic migraine. This point is important for general pediatricians to carefully evaluate before referral to specialists, i.e., pediatric neurologists or pediatric headache specialists. Similar to adults, migraines in children can appear as episodic and chronic, with or without auras [2]. An aura is defined as a transient focal neurological phenomenon that occurs before or during a headache. It develops gradually over several minutes and normally lasts less than an hour. Children may experience one or more aura symptoms, including visual, sensory, or motor symptoms [3]. In general, obtaining a careful and detailed history (including family history, potential triggers, and specific manifestations such as recurrent sickening headaches, occurrence of gastro-intestinal symptoms of nausea, vomiting, diarrhea, and mood changes with headache) is highly recommended, which usually pays off in terms of optimal management later.

Migraine pathophysiology is a complex matter and is only partly understood [4]. One of the mechanisms underlying migraine pathophysiology, in relation to the topic of this review (mood and migraine), is the serotonergic system [5]. The proposal of serotonin biology in migraine is driven from the evidence of changes in serotonin (5-HT) metabolism and in the processing of central 5-HT-mediated responses. Overall, a central neurochemical imbalance with an altered serotonergic disposition has been suggested. Earlier studies have shown lower levels of serotonin, while later PET studies have provided evidence that migraine patients have high levels of serotonin in the brain [6]. The exact path of events related to altered serotonergic neurotransmission is not fully understood, but it has been proposed that 5-HT could influence trigeminovascular nociceptive pathways in migraines [7]. Interestingly, serotonin appears to influence brain function, including mood [8]. However, we do not know how adolescent and pediatric migraines differ, as most of the relevant literature comes from studies on adults.

Migraines occur both in children and adolescents [9] and pose a health problem in this population [10]. Estimates in the literature for affected adolescents and younger children are different [9,11,12], but overall, the occurrence has been estimated between 10 and 25% [13], which makes migraines one of the top 5 childhood health issues [14]. It must be noted that this 10–25% prevalence was found relatively recently. Headache is the second most common reason for neurological care, and one of the most prevalent headache disorders in children [15]. The recurrent nature of migraine headaches negatively influences the quality of life in children, and it highly disables their social and school progression. The Pediatric Migraine Disability Assessment (PedMIDAS) [16] is a tool that helps in determining disability in children with migraine.

In spite of the high prevalence and impact of migraine among the pediatric population, optimal care has progressed slowly. Acting early and properly is important to reduce risks of migraine in adulthood. It has been reported that about half of children with migraine continue to have migraines in adulthood [17]. This in turn may cause a long-term negative impact on life quality and productivity. Transformation changes may also occur when migraines move from childhood to adulthood. In a longitudinal study [17] that followed children with migraine for 40 years, it became evident that the onset of migraine occurrence was at the age of 6 years old on average. 62% of the children became migraine-free for at least 2 years during puberty or in young adulthood [17]. However, 33% of these children experienced a return of regular attacks after 6 years of being migraine-free on average [17]. Of the original 73 children, 60% experienced migraines for as long as 30 years. Among those, 23% did not experience one year without migraine [17]. 52% of the participants who became parents had at least one child who also developed migraine [17]. Pediatric migraine not only affect children but also their families. Major disruptions occur in the lives of pediatric patients with migraine, and one of these is school functioning [18]. Nevertheless, this period is also an important period of psychosocial development for children. Parents need to be aware of the situation and seek medical care and consultation to help affected children return to their normal activities quicker. Family members and school teachers are also encouraged to gain a sufficient understanding about children with migraine, in order to help when they are away from home and their parents. Several infographics and hubs are available for reliable information and recommendation in different countries around the globe.

### 1.2. Mood

A part of emotional rhythm is mood. Changes in daily mood are called mood swings, and environmental factors such as stress can often trigger these changes [19,20,21,22,23]. Other factors have been listed to change a daily mood, including poor sleep, tiredness, news, environmental factors (e.g., pollution, light, and noise), weather, hormonal changes, nutrition, and medication side effects. Monitoring mood may help in the identification of causes and the related behavioral responses, thoughts, and actions. Small children and adolescents might not be able to communicate their mood as adults can, and their reactions might also be different. Therefore, communication with this population and assessments of their daily activities seem important in the context of mood and mood swings. Parents play an essential role, but teachers, caregivers, and consultants can also help by monitoring daily feelings, activities, academic progress, and relationships with peers and others, such as teachers.

Tracking emotion has been one of the widely used ways to understand the nature of mood swings, their origin, and a way to cope with or manage them. A mood diary acts as a useful instrument for emotion-tracking and coping with emotional issues. Digital tracking devices, with analysis interfaces, and easy-to-use applications are currently the most popular tools. A long list of smartphone applications exists for mood tracking from a simple color-coded application to a more complicated and sophisticated application to record functionality, links with energy, and stress level, together with weight, nutrition, medication, and exercise tracking.

It has been recognized that mood changes might be a sign of a more serious medical illness (e.g., depression, anxiety, bipolar disorders, and borderline personality disorder) [24,25]. Patients with mental health issues and psychotic disorders have reported 10-times higher mood swings compared with the general population [20]. The risk of suicide is two times higher in individuals with mood swings [26].

## 2. Methods Used to Study Mood in Children with Migraine

### 2.1. Migraines and Mood in Children

The reaction of small children to migraine might be presented through mood swings, anger, sleep disturbances, violated eating, refraining from socialization, being less active or hyperactive, or abstinence from school. Recurrent headaches can lead to depression and internalization, which often occur in adolescence [27]. Clinical symptoms of depression in children with migraine appear to be different from those in adults [28]. For example, feelings of humiliation, shame, desperation, loss, blame, inadequacy, and sadness might be expressed by an affected child [28]. These facets must be considered for the evaluation of symptoms in children with migraine.

Response to pain might be influenced by catastrophizing, and this critical variable can determine a child’s understanding and adjustment in response to pain [29]. 92 adolescents (13–18 years), including subjects with episodic migraine (n = 40) and healthy controls (n = 52), participated in a study where the Adolescent/Adult Sensory Profile (AASP), the Pain Catastrophizing Scale for children (PCS-ch), and PedMIDAS for children with migraine were determined [30]. Findings from this study [30] showed higher pain catastrophizing levels in the migraine group, where rumination and helplessness were elevated [30]. Therefore, it is valuable to investigate if children and adolescents with migraine have anxiety, depressive mood, or other forms of psychopathology [30,31]. Based on that, offering coping strategies to deal with pain perception seems reasonable to optimize treatment outcome and quality of life. Coping strategies might help affected children with better social, academic, and personal adjustment [32,33].

El-Heneedy and colleagues [34] showed that increased anxious-depressed symptoms among school children with migraine (SCMs) were positively correlated with disabilities following repeated episodes of migraine [34]. In parallel, SCMs showed competence and behavioral problems with increased depressive symptoms, social problems, somatic issues, and difficulties with attention. These consecutively impaired SCMs in their free-time and social activities [34]. Other studies [35,36] have also found increased anxiety and depression in children with migraine. A higher incidence of mood and anxiety disorders was reported in children with migraine by Dyb et al. [37]. Among children and adolescents with migraine, an association of behavioral and internalization symptoms with headache frequency has been found [38,39]. One study [40] examined the prevalence and temporal associations between migraine and daily mood. In this study, 69 children (50 females, 19 males) with migraine (7–12 years), together with their parents participated and provided information about the headaches, emotions, and behaviors in the children and their quality of life [40]. Headache occurrence, duration, severity, mood, daily issues, and medications were recorded once a day for two consecutive weeks [40]. In this study [40], mood was determined by using the facial affective scale (FAS), which is a visual representation of the negative and positive effects of pain and discomfort [41]. Findings demonstrated that worse mood was associated with the a more severe and longer same-day headache [40]. One day’s mood was not associated with the following day’s headache, and one day’s headache was not associated with the following day’s mood [40]. Taken together, these findings [40] suggest that mood changes might be correlated with headache within a given day. Future studies are required to explore the relationship between headaches and mood.

Another study [42] measured triggers of headache in children and adolescents. Self-reported stress was the most reliable predictor for the onset of headache. In addition, when children underwent experimental stress, those with migraine experienced altered physiological arousal and longer recovery compared with healthy controls [43]. In this study, children reported a higher number of hassles on the days they had a headache. They also reported that their negative mood could provoke a headache with a longer duration and a higher severity [43]. Collectively, it seems that a higher number of stressful events might predict headache occurrence and negative emotional response. However, the relationship might be more complicated than has been found. Future studies with close monitoring through an electronic daily diary can reveal if a temporal relationship exists between mood and headaches within a short (one day) or longer duration (several days).

### 2.2. Migraine Phases and Mood in Children

The understanding of migraine in pediatrics has improved as more information has become available about the characteristics and associated symptoms of this special population [3,44]. One of the major milestones has been the recognition of prodrome, aura, and postdrome symptoms and their alterations at the age when children with migraine are in pre- and post-puberty periods [45]. Similar to adults, four phases of a typical migraine attack might be present in children [4] (Table 1). In adults, the prodrome and postdrome are less investigated compared with the headache phase [46,47]. The aura phase corresponds to a very precise and defined neurobiological situation, called cortical spreading depression, which cannot be equated with psychosomatic symptoms. Fewer studies have examined these symptoms in children and adolescents [45,46,47].

In 2016, Karsan et al. studied 100 patients with chronic migraine and found that mood changes, neck stiffness, and fatigue [44] were the most common prodrome symptoms, while neither age nor sex influenced the prevalence of these symptoms. Another study in 2019 [45] used a questionnaire and found that, among 103 children and adolescents with migraine, 67% reported a prodromal symptom. Face changes, fatigue, and irritability were most frequently reported. Postdrome symptoms were reported by 82%. Ocular pain, somnolence, visual disturbances, food craving, and thirst were the most reported symptoms [45]. Fatigue and mood changes were also found as the most common symptoms among 176 patients (an average age of 12 years) [48], where 42% had a premonitory symptom. The results were also independent of age and gender. Anxiety and migraine with an aura were positively correlated in this study [48].

A small study [49] with 19 children with migraine investigated the short-term evolution of pediatric migraine during puberty [49]. The authors also explored whether evolution could influence the quality of life of those children. The researchers of this study [49] obtained the medical history, the migraine characteristics, and the lifestyle characteristics at baseline and after 2 years, and had a telephone conversation for follow-up [49]. The findings showed that dizziness, vertigo, mood changes, confusion, and allodynia were the most common symptoms at the 2-year follow-up [49]. Sleep disturbances showed up as a significant trigger [49]. Taken together, this study [49] provided evidence that prodromal symptoms increased in pediatric migraine, and some trigger factors become more prevalent, namely sleep disturbances. We still need to characterize pre- and post-symptoms [45] that can be improved by prospective designs and using electronic diaries. This may allow us to identify an accurate prevalence, reproducibility over age ranges, and the probability of predicting a headache attack [45]. Developmental factors may play roles in features of pre- and post-symptoms over a given age span (pre- to post-puberty). For example, menstrual migraines that occur only in girls have been studied more than puberty-related effects on boys. Understanding the phenomenology of these sex- and age-evolving symptoms may open new ways of investigating complex interrelations between the prodrome, aura, headache attack, and postdrome in pediatric migraine. This may eventually lead to recommendations and guidelines specific to age (or other factors) to personalize treatment and reduce the negative impact on children’s lives and school performance [45].

### 2.3. Migraine and Psychiatric Comorbidity in Children

Comorbidities must be determined in headache patients. Indeed, it has been found that psychiatric comorbidities interfere with prognosis, the identification of risk for transitions from episodic to chronic conditions, and variations in treatment response, and lead to high costs and a low quality of life [10]. In adults with migraine, psychiatric comorbidities have been studied [11,12], and several psychiatric disorders that co-occur or co-exist with migraine, including anxiety, depression, panic, bipolar, and obsessive-compulsive disorders, have been identified [13]. Hence, an association between mood and anxiety disorders and pediatric migraine [50] has been proposed, but this association is less investigated [51,52,53].

A longitudinal 4-year follow-up study in Norway investigated symptoms of anxiety and depression in adolescents with migraine, and found that having anxiety and depressive symptoms increases the odds of reporting migraine [54]. Another longitudinal study in the US found that major depressive disorders predict the recurrence of headaches [55]. Collectively, these findings suggest that mood and anxiety might be among the risk factors for pediatric migraine, regardless of geographical location. On the other hand, children and adolescents with migraine also have increased anxiety and depression [56]. Dysthymia or depression has been frequently diagnosed in children and adolescents with migraine [8]. Mood problems are commonly seen with chronic migraine [57], which can occur pre- or post-headache. A recent review [58] shows that depression is highly prevalent in pediatric patients with chronic pain. Maintenance of this co-existence has been proposed to be a result of neurobiological abnormalities, influenced by personal traits or other factors such as sleep disturbances, emotion, and behavioral characteristics [58]. A conceptual framework presenting the co-occurrence of chronic pain and depression in youth has been proposed, where factors relating to both the parents and the child can cause chronic pain and depression. In this context, stress has been highlighted as a factor posing a direct impact on the pathogenesis of both conditions, while also influencing both parents- and children-related factors [58]. This concept might be applicable in pediatric migraine with comorbid depression in children. Other comorbid symptoms, including sleep difficulties, dizziness, anxiety, mood, muscle pain, abdominal problems, back and neck pain, and joint-muscle pain, are often reported in children with migraine. Dizziness, feeling weak, nausea, blurred vision, or loss of vision have also been noted [58]. Further studies can potentially uncover how these factors influence migraine in children, and how migraine influences those factors.

Another important angle is that most studies primarily focus on the negative mood changes with migraine, which is appropriate, as it occurs most commonly. However, elevated mood can also be seen. This was reported in the 17th century by Thomas Willis [59], who described his historical headache patient enjoying all the pleasures of life excessively in the days prior to her severe headaches. Elevated mood occurs in the prodrome that may occur in the form of agitation and striking out at others during the headache. Rarely, elevated mood can be seen in the postdrome in the form of an elevated energy level. These data are from adult populations, so elevated mood in children requires further investigation to identify its form and impact [45].

### 2.4. Proposed Mechanisms Underlying Co-Occurrence of Migraine and Psychobiological Response

A psychobiological adaptation might be present in response to migraine in children. Instead of the expression of an uncomfortable condition, a psychological issue, or emotional disturbances, children tend to show physical symptoms [60]. It has been postulated that, during a migraine, the ability to balance homoeostatic changes when facing various stressors seems lacking or impaired. This has been termed as a maladaptive stress response [61,62]. According to this concept, the body mediates environmental stressors and psychological factors, and the development of headaches can be a response to sensory stimuli or physical or emotional stress, hence an adaptive defense [63]. One study [64] investigated a group of children with migraine in comparison with another group of children with chronic musculoskeletal pain. Both groups had vomiting, nausea, perceptual disturbances, and internalizing behaviors. Based on these findings, the investigators proposed that personality and behavioral manifestations in children in general might be related to the experience of chronic pain and not specifically to migraine [64]. This view is in line with the high prevalence of headaches among children when a transition occurs from pre-school to school [65]. The transition for a child is considered a load of stress, which may result in headaches or their worsening as a response [60]. Stress can lower the threshold of an individual to a migraine attack and provoke biological alterations; it can also precipitate the illness. Indeed, hypersensitivity to pain has commonly been reported in migraine patients, which might also be related to stress [66]. Migraines have been strongly associated with depression and anxiety [67]. Depression is known to increase the risk of suicide. Young adolescents are at risk for suicide or suicidal thoughts [68], even if they do not have a depressive mood. Therefore, screening and consultations for this population seem beneficial [69].

Mood and anxiety symptoms occurring with migraine in children [53,70] further reduce these children’s function [71], quality of life [72], and social relationships [73]. A recent review [69] has presented a diverse range of factors, which may influence learning and academic performance in these children. Learning difficulties might be due to headache itself, other psychiatric symptoms, or a combination of both [74]. Sleep disorders were also found associated with headache, mood disorders, and learning [74].

A high incidence of psychiatric and sleep abnormalities have been found in school-aged children with migraine [34]. The most common psychiatric disorders were anxiety and depression, withdrawal depressed symptoms, social issues, and problems with attention. Sleep disturbances were present as low sleep quality, excessive daytime sleepiness, and decreased total sleep time, among several other abnormal sleep indicators [34]. Considering sleep hygiene may help patients recover from the vicious sleep-headache cycle, where children are affected by a nocturnal attack that disturbs sleep, and sleep disturbances exacerbate headaches [75].

Children are affected by stressors and environmental factors that might be different from adults. Therefore, it is valuable to investigate further the most common and important environmental factors that can enhance the risk of migraine attacks or the co-occurrence of headache with other psychological issues. School life and educational performance affect children with migraine differently from adults in academia [74]. Understanding stressors in these years of life for children with migraine can assist in the implementation of plans. Interestingly, it has been shown that the burden of migraine in children changes with the schooling schedule, with the start of the school year being the worst period [76]. This also reflects in children with a better mood in the summer and a worse mood in the wintertime.

## 3. Considerations for Treatment Strategies in Children with Migraine

A relationship between mood and migraine exists and is influenced by several factors (Table 2).

**Figure 1:**
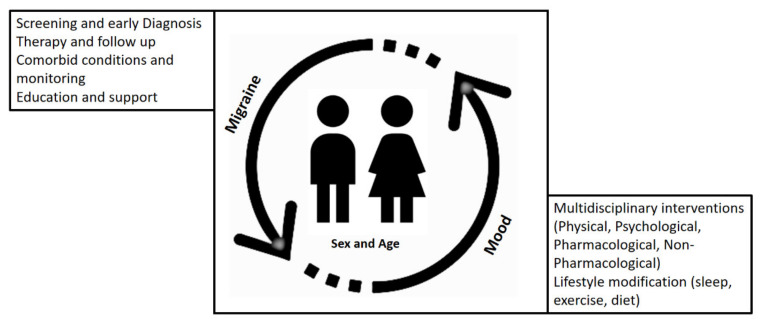
Mood and migraine relationship and points for treatment strategies.

Several important points for treatment strategies have been considered that can help to obtain an optimal outcome when mood and migraine are interacting in affected children (Figure 1).

Migraine by nature is a recurrent condition and negatively affects the psychological state and social status of children who have them [77]. This in turn negatively affects the quality of life and school performance. Therefore, early diagnosis and proper treatment are essential [78,79] to prevent complications of long-term migraine conditions [80,81].

Evidence-based treatment guidelines [82] have been provided for pharmacological and non-pharmacological interventions. Behavioral interventions [83], including education [84], relaxation, biofeedback, and cognitive-behavioral treatment (CBT), have yielded positive results for migraineurs [85]. More studies have been done for adults with migraine [85,86,87], but data on similar strategies for managing migraine in children and adolescents are limited [13,88,89,90,91]. According to Faedda et al. [83], behavioral therapy consists of three components: Adherence to treatment, lifestyle adjustment, and psychological intervention. Educating the affected child and their parents is important and can be aided by closely following verbal and written instructions of treatment plans that can be provided. It can also help reduce the helplessness and fear of affected children and enable their daily function. The establishment and practice of healthy lifestyle habits are highly crucial in pediatric migraine cases. It has been shown that lifestyle habits interfere greatly with medical outcomes [92]. In this line, keeping a headache diary has been used as a useful way to keep headaches under control and to avoid triggers such as stress, sleep, weather, light, odor, sound, and diet [83,93,94]. In children and adolescents, psychological treatments are essential elements of the multidisciplinary, bio-psychosocial management of primary headache disorders [95]. Perhaps this stems from the observations that headaches are commonly associated with psychiatric comorbidities (depression, anxiety, and attention-deficit/hyperactivity disorder) [96]. Relaxation techniques, tools, and recommendations for stress reduction, increasing physical activity, and other psychological interventions have been proposed and used, which all have one thing in common: The active involvement of patients in treatment plans. Sieberg et al. [95] classified psychological treatments for primary headache in three categories: Relaxation skills [97], biofeedback [98], and cognitive behavioral therapy (CBT) [99]. Interestingly, non-pharmacological interventions, e.g., adding CBT to amitriptyline treatment, often improve pharmacological responsiveness as well for children and adolescents with chronic migraine [100].

### 3.1. Pharmacological Treatments

Two recently updated guidelines are available for the acute [82] pharmacological treatment of migraine in children and adolescents and for prevention therapy [101]. However, rigorous studies that examine every aspect of treatment recommendations, including lifestyle, non-pharmacological treatments, and combinations, are needed [102]. In addition, Penzien et al. [97] have also made recommendations pertaining to behavioral interventions for migraine, such as relaxation, thermal biofeedback, electromyographic biofeedback, cognitive CBT for migraine prevention (Grade A evidence), and behavioral therapy combined with preventive therapy (Grade B evidence) [82,101].

Ibuprofen, acetaminophen, sumatriptan, and zolmitriptan nasal spray are among the most studied drugs for the acute treatment of migraine in children [48]. Ibuprofen and sumatriptan were found significantly effective in the acute management of childhood headaches in the emergency department [103]. Preventative therapies are required in a subset of children with migraine (20–30%). Antiepileptic drugs, antidepressants, antihistamines, and antihypertensive agents are used for the prophylactic management of chronic migraine [79]. Based on the individual needs of children with migraine and psychological co-morbidities, e.g., mood disorders or other comorbid conditions such as asthma and diabetes, an optimal drug can be chosen by a pediatric neurologist or headache specialist. In this regard, the specific unwanted effects of a chosen drug and their impact on a patient’s life (e.g., weight gain or sedation) must be carefully evaluated. Drugs that can have dual effects on mood disorders and headaches are preferred when children with migraine are affected by the co-existence of these conditions. New pharmacological treatments (e.g., CGRP antagonists) for migraine are available that might also be considered safe for children depending on their age and condition.

### 3.2. Non-Pharmacological Treatments

Several triggers have been identified for pediatric migraine. According to a recent review, stress, a lack of sleep, weather, video games, intense noise, and light have been highlighted [13].

Regular hydration and a balanced diet, as well as good sleep hygiene with an adequate bed and regular waking times to allow for sufficient sleep time, are popular lifestyle modifications [104] often recommended to children with migraine and to their parents. Strikingly, only 17% of adolescents stay sufficiently hydrated during a given day [4]. It was found in another study that 10% of children have experienced migraines due to dehydration, and 85% experienced migraine attack relief from rehydration [46]. Food triggers should be identified and avoided; however, the general agreement currently is the maintenance of a balanced diet. Interestingly, diet and mood [105] are interrelated, and a bidirectional relationship between diet and migraine has been proposed [106,107]. Therefore, mood and food deserve further investigation in pediatric migraine.

Macronutrients have shown a preventive role in migraine [108]. CoQ10 level in children has been found below the reference range in 33% of children with migraine [109]. Supplementation with CoQ10 improved PedMIDAS scores and headache frequency [109]. Another trial by Slater et al. [110] with CoQ10 supplementation also presented an improvement in the frequency of headaches within 4 weeks. Vitamin and folate supplementation was investigated in adult patients with migraine and showed efficacy [111], but there are no placebo-controlled studies in children. The effect of vitamin D3 supplementation in children is not known, but adolescents have been found to be deficient in vitamin D3 (24.1%) [112]. Due to the high placebo effect (reported as 30–60%) for the prevention of childhood migraine, an accurate evaluation of CAM effectiveness is not possible, and a recent review [113] shows that current literature is still premature to confirm the effectiveness of complementary and integrative medicines (CIMs) for pediatric migraine.

Based on Yamanaka et al.’s review [13], various sleep disorders, including insufficient sleep, sleep bruxism, co-sleeping with parents, snoring, daytime sleepiness, difficulty with falling sleep, night walking, and difficulties in sleep maintenance, have been identified in children with migraine. Good sleep hygiene, defined as a regular bedtime, an avoidance of screens before bedtime, and a similar schedule on weekends, has reduced headache frequency in children [114]. Regular exercise is beneficial for migraine prevention [115], and it is proposed that this might be due to its effect on increasing endogenous beta-endorphins [116]. Lower levels of beta-endorphin have been found in the cerebrospinal fluid of adult migraineurs [116]. The role of sleep [117] and exercise [118] in treating mood disorders has also been reported. Therefore, sleep hygiene and regular exercise seem beneficial for mood and migraine in children.

As mentioned earlier, relaxation training and biofeedback proved beneficial for children with migraine [89]. Biofeedback reduces the pain and frequency of headaches [119]. Blume et al. [120] analyzed data from children with chronic migraine who received two or more biofeedback sessions and found a statistically significant reduction in their headache days. It is suggested that feedback training can normalize cortical excitability and relieve headaches [121]. CBT has also been discussed in the literature for many years, and the focus now is to further understand how and why CBT works and to implement it into headache, neurology, and primary care clinics [122,123]. Relaxation apps are available for mobile electronic devices. Biobehavioral guidelines are also under development [124]. Since these techniques and CBT are well-established treatments for managing anxiety and depression in children and adolescents [125,126], CBT-based treatments and relaxation together with behavioral therapy must be considered for the treatment for mood disorders, such as pediatric internalizing disorders [126]. This may be particularly beneficial for children with migraine and mood disorders.

To achieve optimal headache control, many patients are encouraged to follow a multidimensional approach consisting of lifestyle changes and the treatment of comorbid conditions [9]. CBT together with standard antimigraine therapy in pediatrics has shown high efficacy [127]. A biopsychosocial approach seems most effective for the treatment of migraines in children [79,128]. An algorithm considering individualized treatment based on compliance of the pediatric patient, disability, and co-morbidities was proposed by Orr and colleagues in 2018 [79]. This individualized treatment plan can include self-management strategies, acute and preventive therapy, which requires an interdisciplinary team, education, and lifestyle modifications, including improvements in sleep hygiene, pharmacological, psychological, and nutraceutical interventions, and diet changes [79]. Nutraceutical interventions in children and adolescents with migraine are less investigated. Considering the results from the Childhood and Adolescent Migraine Prevention (CHAMP) Study [129,130], the placebo effect of pill-taking seems to be in favor of migraine relief for children. The future awaits the implementation of cost-effective models for the delivery of biopsychosocial approaches for pediatric migraine [79]. Interestingly, many of these non-pharmacological strategies used for migraine have proven effective for mood disorders in children, while also offering an acceptable safety profile [131].

## 4. Concluding Remarks

Migraines negatively influence the quality of life of affected children. Early diagnosis and management decisions are needed to reduce the burden and maximize the treatment outcome. Mood swings can be monitored to help in early diagnosis, reducing disability, and improving children’s emotional functioning. If a mood swing turns out to be a prodromal syndrome, it can be a guide to proper and on-time care, but it can also reflect more serious conditions in children and adolescents, such as comorbid psychological issues (anxiety and depression). Mood swing monitoring can help in reducing factors such as stress and in finding highly personalized coping strategies. Several mood tracking apps are available that can be selected and used based on the age of, and applicability for, pediatric migraineurs. Parents, caregivers, and teachers can play an important role in the identification of temporal patterns of mood swings in children with migraine. Longitudinal studies can determine the evolving nature of this process as children undergo puberty. The underlying mechanisms of the interaction between mood and migraine can also be investigated to discover new therapeutic strategies and targeting. The changes in the characteristics of migraine that occur as a result of the age and sex of an affected child are unique. The developmental aspects of behavior, weight, mood, sleep, activity patterns, coping, and metabolic alterations, in addition to neuroendocrine and stressors, require further attention if optimal prevention and treatment are to be achieved.

## Figures and Tables

**Table 1:** Popular symptoms of four phases of a typical migraine attack.

Prodrome	Aura	Headache	Postdrome
Gastrointestinal disturbances (e.g., constipation or diarrhea) Food craving or thirst Neck stiffness Frequent yawning, Fatigue Mood changes (depression, irritability, anger, and anxiety)	Visual symptoms (e.g., flashing lights, blank spots, and blurry vision) Olfactory or auditory hallucinations Hypersensitivity or reduced sensation Tingling or numbness (e.g., in face or extremities) Difficulty speaking Confusion, dizziness, or vertigo	Head pain that can become bilateral Sensitivity to light and sound Nausea and vomiting Depression or anxiety Dizziness	Feeling tired Confused Mood changes (feeling melancholy or depression) Poor concentration Poor memory

**Table 2:** Factors affecting mood and migraine relationship in children.

Child’s personality traits	Education
Individual child (sex and age)	Monitoring
Children family	Treatment strategies
School and community	Intervention feature
Children communication feature	Stressors
Care system	Adherence
Comorbid conditions	Compliance
Lifestyle (sleep, diet, and exercise)	Placebo effect
Puberty	Nocebo effect
